# Decreased Modulation of EEG Oscillations in High-Functioning Autism during a Motor Control Task

**DOI:** 10.3389/fnhum.2016.00198

**Published:** 2016-05-06

**Authors:** Joshua B. Ewen, Balaji M. Lakshmanan, Ajay S. Pillai, Danielle McAuliffe, Carrie Nettles, Mark Hallett, Nathan E. Crone, Stewart H. Mostofsky

**Affiliations:** ^1^Department of Neurology and Developmental Medicine, Kennedy Krieger InstituteBaltimore, MD, USA; ^2^Department of Neurology, Johns Hopkins University School of MedicineBaltimore, MD, USA; ^3^Department of Psychological and Brain Sciences, Johns Hopkins UniversityBaltimore, MD, USA; ^4^Human Motor Control Section, Medical Neurology Branch, National Institute of Neurological Disorders and Stroke, National Institutes of HealthBethesda, MD, USA; ^5^Center for Neurodevelopmental and Imaging Research, Kennedy Krieger InstituteBaltimore, MD, USA; ^6^Department of Psychiatry and Behavioral Sciences, Johns Hopkins University School of MedicineBaltimore, MD, USA

**Keywords:** dyspraxia, motor planning, event-related desynchronization, autism, praxis

## Abstract

Autism spectrum disorders (ASD) are thought to result in part from altered cortical excitatory-inhibitory balance; this pathophysiology may impact the generation of oscillations on electroencephalogram (EEG). We investigated premotor-parietal cortical physiology associated with praxis, which has strong theoretical and empirical associations with ASD symptomatology. Twenty five children with high-functioning ASD (HFA) and 33 controls performed a praxis task involving the pantomiming of tool use, while EEG was recorded. We assessed task-related modulation of signal power in alpha and beta frequency bands. Compared with controls, subjects with HFA showed 27% less left central (motor/premotor) beta (18–22 Hz) event-related desynchronization (ERD; *p* = 0.030), as well as 24% less left parietal alpha (7–13 Hz) ERD (*p* = 0.046). Within the HFA group, blunting of central ERD attenuation was associated with impairments in clinical measures of praxis imitation (*r* = −0.4; *p* = 0.04) and increased autism severity (*r* = 0.48; *p* = 0.016). The modulation of central beta activity is associated, among other things, with motor imagery, which may be necessary for imitation. Impaired imitation has been associated with core features of ASD. Altered modulation of oscillatory activity may be mechanistically involved in those aspects of motor network function that relate to the core symptoms of ASD.

## Introduction

A key hypothesis of the neurobiology of autism spectrum disorders (ASD) is that cortical excitatory function is not sufficiently balanced by inhibitory forces (Rubenstein and Merzenich, 2003; Rubenstein, 2010). This proposition is supported by converging evidence from mouse model histology (Rubenstein, 2010) as well as the clinical observation of an increased prevalence of epilepsy in ASD (Viscidi et al., 2013). As GABAergic activity in the brain is partly responsible for coordination of neural activity, inhibitory alterations in ASD may lead to altered synchronization, which is postulated by some to be a fundamental cause of the ASD phenotype (Uhlhaas and Singer, 2006), possibly by reducing the “signal-to-noise ratio” of task-relevant information (Markram and Markram, 2010).

Oscillatory electrical activity, as captured on the electroencephalogram (EEG), is a phenomenon molded by inhibitory activity. Specifically, the oscillatory activity seen on the EEG reflects, at least in part, the synchronization of neuronal activity through the action of GABAergic interneurons (Uhlhaas and Singer, 2010; Gaetz et al., 2011), whose firing patterns create rhythm in principal cells (Buzsáki and Chrobak, 1995). Alterations of electrical brain oscillatory activity may therefore be a useful window into the pathophysiology of the inhibitory interneuron system in conditions such as ASD (Wilson et al., 2007; Uhlhaas et al., 2010).

Indeed, there is evidence—though limited—for altered cortical oscillatory behavior in ASD. Much of the evidence to date comes from resting state (spontaneous, task-free) EEG and consists of somewhat disparate findings (Cantor et al., 1986; Dawson et al., 1995; Daoust et al., 2004; Chan and Leung, 2006; Murias et al., 2007; Orekhova et al., 2007; Cornew et al., 2012). The oscillatory activity seen on the EEG is not merely an epiphenominological marker of interneuron function, but is rather directly involved in cortical computation (Başar et al., 2001; Kelly et al., 2006; Fries et al., 2007; Engel and Fries, 2010). We therefore intended to examine the role of potential oscillatory changes in ASD in altered cortical processing. This goal is best achieved when oscillatory measures are directly associated with a disorder-relevant task.

Complex motor control is a useful model for studying cortical network dynamics and behavioral performance in ASD. Motor deficits were noted in Kanner’s (1943) original description of autism, and their study is important not only for their own sake, but also because motor control deficits relate theoretically (Mostofsky and Ewen, 2011) and empirically (Dziuk et al., 2007) to the social and communicative impairments in ASD. The benefits of studying the motor system are practical. Neural substrates of the motor system are relatively well characterized (Uttal, 2012), and tasks involving motor output lend themselves to experimental manipulation and reliable quantification. Our laboratory focuses on praxis, the performance of complex, skilled gestures used in functional skills and communication (Heilman and Valenstein, 2003). Neuropsychological and neurophysiological studies of acquired apraxia and developmental dyspraxia have allowed investigators to interrogate the praxis network, which includes inferior parietal and premotor (frontal) areas (Wheaton and Hallett, 2007). Convergent lesion and physiological studies have shown lateralization to the left (Moll et al., 2000; Wheaton and Hallett, 2007).

The link between praxis and the cardinal features of ASD is both theoretical and empirical. On the theoretical side, current frameworks suggest that the motor system plays an important role in the internal simulation of others’ actions, which allows for understanding of others’ intentions and affective states (Klin et al., 2003; Gallese, 2007; Mostofsky and Ewen, 2011). Empirically, we have shown a relationship between deficits in motor control on the one hand and social-communicative impairment in children with ASD on the other (Dziuk et al., 2007; Dowell et al., 2009).

In order to assess oscillatory modulation during a task relevant to the ASD phenotype, we recorded EEG while subjects performed a praxis task. Specifically, we looked at task-related modulation, relative to baseline, of power in the EEG signal (*event-related spectral perturbations*; ERSP) and hypothesized that children with ASD would show altered ERSP responses in both posterior and central sensors on the left, reflecting the activity of left parietal and frontal premotor praxis-related brain regions. To assess relevance to the ASD phenotype, we examined correlations between physiological EEG measurements and behavioral testing of ASD severity and praxis function.

## Materials and Methods

### Subjects

Analyses included data from 25 subjects with high-functioning ASD (HFA; defined as ASD with at least low-average IQ; (Ghaziuddin and Mountain-Kimchi, 2004) and 33 controls (or *typically developing* participants; TD). As described below, 40 additional subjects’ data were removed from analysis due to poor behavioral performance and/or excessive artifact, as set out in section “Signal Pre-Processing”. The bar for data quality was set high to reduce the likelihood of type I errors. Inclusion criteria relevant to both groups included age between the eighth and thirteenth birthday, a Wechsler Intelligence Scale for Children—IV (Wechesler, 2003) full-scale IQ > 80 (except in the presence of a split of greater than 12 points between Verbal Comprehension Index and Perceptual Reasoning Index, in which case the higher score was >80 and the lower >65) and right handedness [Edinburgh (Oldfield, 1971) and PANESS (Denckla, 1985)]. Edinburgh results were to be between 0.5 and 1. If the Edinburgh was not available, children were asked to do 11 gestures from the PANESS; at least 10 must be have been done spontaneously with the right hand for the subject to qualify for the study. General exclusion criteria were diagnosed neurological or chronic medical condition, severe visual impairment (corrected worse than 20/40) and pregnancy.

ASD diagnosis was conservative and based on DSM-IV (American Psychiatric Association, 2000) criteria, with positive scores on both ADOS (Lord et al., 2000, 2012) and ADI-R (Lord et al., 1994), and clinical confirmation by child neurologist experienced in autism diagnosis (SHM). Children with a known neurological, genetic, metabolic or other etiology associated with the ASD were excluded. All children were assessed with the Diagnostic Interview for Children and Adolescents-IV (DICA-IV; Reich et al., 1997) or Schedule for Affective Disorders and Schizophrenia for School-Age Children (K-SADS; Kaufman et al., 2013); children were excluded from the ASD group if they had any diagnosis other than: (1) ASD; (2) an anxiety disorder (given the extensive comorbidity with ASD as well as the large overlap between obsessive-compulsive symptomatology and ASD symptomatology in particular); and (3) attention-deficit/hyperactivity disorder (ADHD; given the high levels of comorbidity with ASD).

Seventeen participants with HFA also had a diagnosis of ADHD. Co-occurring ADHD was defined by convergence of performance on three measures: (1) meeting diagnostic criteria on either the DICA-IV or K-SADS; (2) obtaining a *t-*score ≥65 on the Connors Parent Rating Scale (Conners et al., 1998) for either the hyperactive/impulsive scale, inattentive scale, or both; and (3) having at least 6 symptoms rated as 2 (often) or 3 (very often) on the DuPaul rating scale (fourth edition; Dupaul et al., 1998) for either the hyperactive/impulsive scale, the inattentive scale, or both. In cases in which one or two measures, but not all three, were positive, the final diagnosis of ADHD was based on DICA/K-SADS and clinical impression from an experienced autism/ADHD clinician and researcher (SHM). Eight subjects with ASD were diagnosed with an anxiety disorder (including obsessive-compulsive disorder, generalized anxiety disorder and simple phobia). Six had no secondary psychiatric diagnosis. Eight participants in the HFA group were on medication (2 dextroamphetamine salts, 5 methylphenadate/dexmethylphenadate, 1 “homeopathic lithium carbonate” + clonidine); stimulant medication was stopped 24 h prior to testing.

Entry into the control group was only permitted for children who did not meet published criteria for ASD on the Social Responsiveness Scale (Constantino and Gruber, 2005) and absence of first-degree family members with ASD. Children were also ineligible for entry into the TD group if they met diagnostic criteria for any disorder on the DICA-IV (Reich et al., 1997), except for simple phobia. No subject in the control group had a psychiatric diagnosis. Seven control subjects were on second-generation histamine blockers for seasonal allergies, inhaled steroids and leukotriene inhibitors and as-needed decongestants.

The protocol was approved by Johns Hopkins Medicine Institutional Review Board. Written consent was obtained from parents and, when possible, from subjects. Otherwise, verbal assent was obtained from subjects.

### Task and Behavioral Monitoring

The task was based on that used by Wheaton et al. (2009), as described in a previous study (Ewen et al., 2014). Modifications to Wheaton’s original paradigm were designed to mitigate confounds due to known working memory and linguistic deficits in ASD. The paradigm consisted of pantomiming the use of 10 common tools (scissors, spoon [to stir hot chocolate], ice cream scoop, doorknob, pencil, screwdriver, hammer, paintbrush, key, chalkboard eraser). Prior to the recording, the participants demonstrated the correct use of each of the tools to the experimenter, who remained next to the subject for the duration of the task.

**Figure 1:**
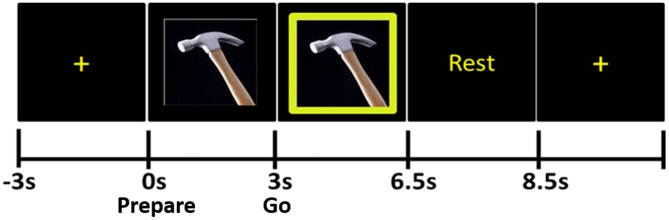
**Praxis task.** The tool photograph that appears immediately after the fixation cross is the *Prepare* stimulus, during the presentation of which subjects do not move. When the green frame appears around the photograph (*Go* stimulus), subjects pantomime the use of the tool.

The stimuli were presented using *eevoke* software (Advanced Neuro Technologies [ANT], Netherlands). During the pre-stimulus portion of each trial, subjects fixated on a cross at the center of the computer monitor for 4 s (Figure 1). Next, the *Prepare* stimulus appeared: this was a photograph of one of the ten tools; participants were instructed not to make any movements during this time. After the *Prepare* stimulus remained on the screen for 3 s, a green box appeared around the photograph of the tool; this was the *“Go”* stimulus. The *Go* stimulus remained on the screen for 3.5 s, during which time the subjects were to pantomime the use of the tool with their right hand until the word “Rest” appeared. “Rest” lasted 2 s and was replaced by the fixation cross for the next trial. Six blocks of 20 trials each (120 trials total) were recorded in each subject.

To ensure adequate behavioral performance, a psychological associate, trained in the conduct of psychological and motor research in children with developmental disabilities and determined to be research-reliable in the offline coding of the pediatric adaptation of the Florida Apraxia Battery (FAB; Mostofsky et al., 2006), monitored the performance of the subject and used a button to mark the electronic record whenever a subject started the movement early (before the onset of *Go*) or late (>1000 ms following the onset of *Go*), finished early or made errors in the movement/no response. Movement errors were coded by the same stringent criteria as in Mostofsky et al. (2006) and other publications examining dyspraxia in ASD (Dziuk et al., 2007; Dowell et al., 2009; MacNeil and Mostofsky, 2012). All sessions were video recorded, and another experimenter examined the video of each trial by the same criteria. Only movements that were initiated within the allowable time-frame and were error-free by FAB criteria, by both raters, were included in the EEG analysis.

### Recordings

**Figure 2:**
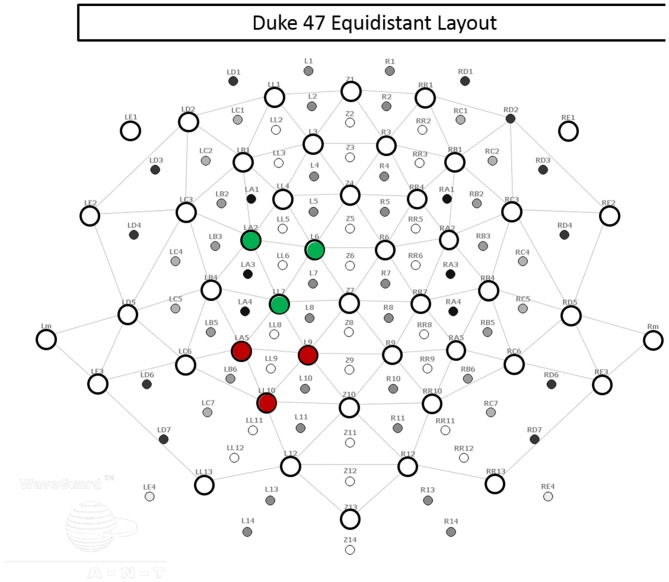
**Electrode layout.** The 47 electrodes used in recording are represented by open circles. Electrodes within the left central regions of interest (ROI) are indicated in green, and electrodes within the left posterior ROI are indicated in red.

Using an Advanced Neuro Technologies *asa-lab* system (Netherlands) and elastic mesh *Waveguard* cap (Figure 2), EEG data were recorded from 47 sites covering the whole scalp with approximately uniform density. The recording was performed in DC mode, with a 512 Hz sampling rate and an anti-aliasing filter with a 138 Hz cut off. Each channel was referenced to an average of all channels during recording. The electrode cap used active cable-shielding technology. Electrode impedance was kept below 5 kΩ in all channels.

### Signal Pre-Processing

To minimize the spatial blurring due to volume conduction, signals were converted from average reference to current source density (CSD) estimates, computed using the spherical spline algorithm (Kayser and Tenke, 2006). In the CSD montage, vertical electro-oculograms (VEOG) were recorded from frontal channels whose locations were designed specifically to capture eye blinks. Eye blink correction was then performed using a principal component analysis (PCA) method that models the brain signal and artifact subspaces (Ille et al., 2002).

CSD data from each trial were epoched by time-locking to the onset of the *Prepare* stimulus. Epochs were created with a pre-stimulus (baseline) length of 2 s and post-*Prepare-*stimulus-onset (trial) length of 6 s, for a total length of 8 s. In the remainder of the article, event-related timing is referred to in reference to the onset of the *Prepare* stimulus.

Epochs with horizontal eye movement artifacts, recognized as step-like potentials of opposite polarity in channels RE1 and LE1 (electrodes located 1–2 cm lateral to external epicanthi), were removed if the amplitude exceeded a threshold defined individually for each subject (based on analysis of definite lateral eye movements). Muscular and movement artifacts (such as jaw clenches) were identified by visually inspecting the signal and also by video verification. Epochs with these artifacts were removed from analysis.

We excluded from all analyses all subjects who did not finish all six runs of the experiment. We further excluded any subject for whom more than 50 percent of trials had to be excluded due to errors in behavioral performance, as described in section “Recordings”. In total, 18 subjects were removed from the HFA group and 14 from the TD group for insufficient behavioral performance. Following pre-processing of the EEG, we next excluded any subject who failed to have more than 50 percent of trials that were both artifact-free and behaviorally correct. Four subjects were removed from each group for insufficient trials after EEG pre-processing. All excluded subjects were removed prior to ERSP analysis.

### Time-Frequency Decomposition

Time-frequency decomposition of the CSD data was accomplished using Morlet wavelet decomposition in EEGLAB (Delorme and Makeig, 2004), with a window size of 512 points and frequencies of 0.5–30 Hz. Each time-frequency point of the active portion of the ERSP analysis (i.e., following the onset of the *Prepare* stimulus) was calculated as a *z-*score, based on the baseline period.

For each trial, at each frequency, all measurements of signal energy during the 2-s baseline interval were aggregated into a distribution. Next, the ERSP value at each time-frequency point during the active phase (0–6 s, relative to the onset of *Prepare*) was calculated as a *z-*score value relative to the baseline distribution for the same trial, at the relevant frequency. We did not remove phase-locked activity prior to ERSP calculation.

### Channel Selection

We next selected relevant channels based on the known anatomy of the praxis network and on precedent from previous EEG studies of praxis (Wheaton et al., 2005b; Ewen et al., 2014). We selected electrodes in scalp regions superficial to the central (premotor/primary motor) and parietal areas that constitute the principal nodes of the praxis network. Although the article from Hallett’s laboratory (Wheaton et al., 2005b) used a different electrode layout, and the present study used a more parsed sensor array compared with our prior study (Ewen et al., 2014), the electrodes selected in the current study represent sensor locations that overlap with those in the previous works. As described below (see “ERD Quantification” Section), we used regions of interest (ROI) to characterize the amount of ERSP in each individual’s central and parietal areas. The rationale for this approach was that using multiple channels in a ROI, rather than a single channel, would mitigate the variance in correspondence between channels and brain generators between patients. Further, the effects of volume conduction, though already limited through the use of CSD spatial filtering, would be further decreased by averaging out activity peripheral to the ROI.

### Frequency Selection

**Figure 3:**
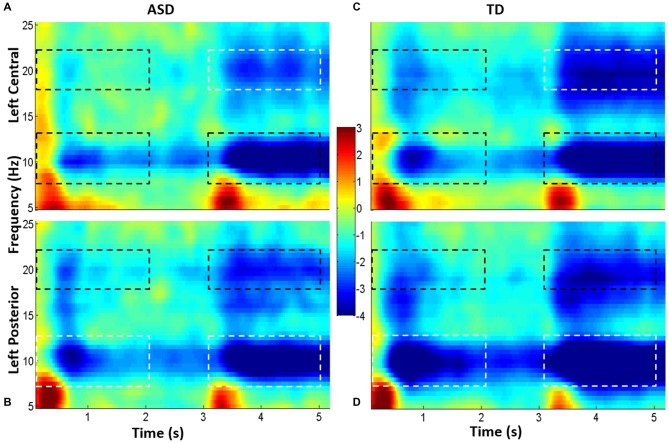
**Grand average spectrograms from children with high-functioning ASD (HFA; A,B) and typically developing (TD) controls (C,D).** Cool colors represent event-related desynchronization (ERD), and warm colors represent event-related synchronization (ERS). ERD is indicated in unitless *z*-scores. Time is indicated along the *x*-axis (in seconds), and frequency is indicated along the *y-*axis (in Hz). The top spectrograms **(A,C)** represents the left central ROI, and the bottom, the left parietal ROI **(B,D)**. Dotted boxes indicate time-frequency regions that were averaged and compared between groups; regions that were statistically significantly different between groups are in white boxes; those which were not different are in black boxes.

Using the grand average spectrogram for each ROI, for each group (i.e., four in total), we defined the frequency ranges of interest (Figure 3) by examining delimited regions in the frequency axis of ERSP. There was great consistency in relevant frequency bands between central and posterior spectrograms, and these frequency ranges were consistent with traditionally described frequency bands: theta (2–8 Hz), alpha (7–13 Hz) and beta (18–22 Hz). Of note, the beta band selected comports with that used by Wheaton et al. (2005a, 2009) in a series of EEG articles examining praxis.

### ERD Quantification

For each subject, we quantified mean event-related desynchronization (ERD) magnitude separately in eight time-frequency-topography windows: two time ranges (*Prepare* = 0–2 s, and *Go* = 3–5 s) × the two frequency ranges (alpha and beta) × two ROIs (left central and left posterior ROI, averaging over the three channels in each ROI; Figure 2). For alpha and beta separately, we performed a Group (ASD, TD) × Task (*Prepare*, *Go*) × ROI (LP, LC) repeated measures ANOVA, with Task and ROI as the within-subjects factors and Group as the between-subjects factor, using SPSS. *Post hoc*, we examined for group differences by using Welch’s *t*-test (no assumption of equal variances; two unmatched samples, two-sided) in each of the eight time-frequency-topography windows.

We additionally measured mean baseline EEG power in each ROI, in each frequency band (alpha and beta), using the time window of −2 to 0 s relative to the onset of the *Prepare* stimulus. We compared the samples from each group using Welch’s *t*-test (no assumption of equal variances; two unmatched samples, two-sided) as well as JZS Bayesian Factor and Information Bayesian Factor.

### Additional Behavioral Measures and Comparisons with Physiological Measures

*A priori*, we defined a set of clinical and behavioral tests we believed could have a theoretical relationship to the physiological effects being measured. These included the ADOS, SRS and a pediatric modification of the FAB (Gonzalez Rothi et al., 1997; Mostofsky et al., 2006), which is a more comprehensive praxis competence assessment than the task used during EEG and better able to discriminate levels of ability amongst subjects. The modified FAB examines a wide range of praxis-relevant motor behavior, including tool use gestures, communicative gestures and meaningless gestures, under the conditions of gesture-to-command, gesture-to-imitation and performance of tool use gestures while holding the actual tool.

Subjects’ results on the praxis battery were compared between groups (using Student’s *t*-test). We then assessed correlations, separately within the two groups, between those ERSP measures found to be different between groups on the one hand and praxis, ADOS and SRS measures on the other. Correlations were assessed using Pearson *r*-tests. Some subjects were tested with the ADOS-G and others with the ADOS-2, so we adjusted ADOS-2 severity scores to be comparable to ADOS-G scores (Gotham et al., 2009).

## Results

### Participants

Average age for children with HFA was 10.7 ± 1.4 years; for control children, 10.52 ± 1.3 years; two-sided Student’s *t*-test *p* = 0.62). Twenty four percent of HFA subjects were female; 24% of TD subjects were female (Fisher’s exact test *p* = 1). Full-scale IQ was not different between groups (mean FSIQ for *TD* = 115.6, and for HFA = 109.3; *p* = 0.095). To further examine for group differences in IQ, we used two Bayesian analyses, each with two priors. The Scaled JZS Bayes Factor = 1.15, and Scaled-Information Bayes Factor = 0.825 (Rouder et al., 2009), both failing to provide evidence of group differences in IQ. Within the HFA group, the mean ADOS score was 12.5 (range: 8–20).

### Event-Related Spectral Perturbations

**Figure 4:**
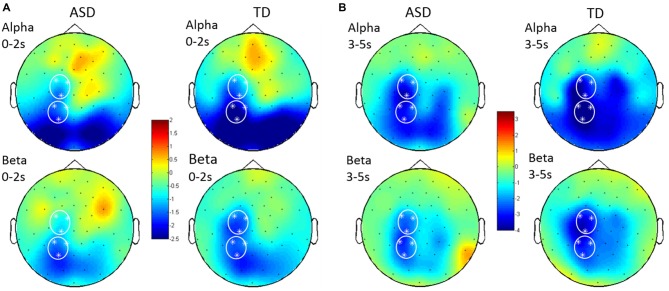
**Topographical distribution of alpha and beta ERD in *Prepare* (A) and *Go* (B).** Alpha ERD (in unitless *z*-scores) was seen in occipital and parietal scalp regions following *Prepare*, and beta ERD was seen principally in parietal regions during this phase. Following *Go*, both alpha and beta ERD were seen in parietal and central (motor/premotor) regions. Signals were analyzed from left central and left posterior ROIs. Grand average topographical plots from children with HFA on the left and typically developing (TD) children on the right.

Subjects from both groups showed a characteristic pattern of ERSP (Figures 3, 4). Specifically, brief event-related synchronization (ERS; task-related increase in power) was seen in the delta/theta band (2–8 Hz), while sustained event-related desynchronization (ERD; task-related decrease in power) was seen in both alpha (7–13 Hz) and beta (18–22 Hz). We examined the magnitude of alpha and beta ERD.

In the alpha band, the RM-ANOVA showed a significant effect of task (*F*_(1,56)_ = 89.686, *p* < 0.0001, $\eta_{p}^{2}$ = 0.616) and ROI (*F*_(1,56)_ = 5.137, *p* = 0.027, $\eta_{p}^{2}$ = 0.084). There were also significant interactions between ROI and Group (*F*_(1,56)_ = 11.466, *p* < 0.017, $\eta_{p}^{2}$ = 0.097), and between ROI and Task (*F*_(1,56)_ = 6.221, *p* = 0.016, $\eta_{p}^{2}$ = 0.1). In the beta band, we found a significant effect of task (*F*_(1,56)_ = 74.797, *p* < 0.0001, $\eta_{p}^{2}$ = 0.572) and a significant interaction between ROI and task (*F*_(1,56)_ = 5.598, *p* = 0.021, $\eta_{p}^{2}$ = 0.91). The full ANOVA tables are presented in the Supplementary Material (see “Supplementary Data 2”).

*Post hoc* *t*-tests showed children with HFA had, on average, a decreased magnitude of alpha ERD in the posterior ROI (29% difference in sample means, *p* = 0.0045) during *Prepare.* During *Prepare*, beta ERD showed near-significant differences (25% difference in sample means; *p* = 0.06).

During the *Go* phase, a decreased magnitude of left parietal alpha ERD was again seen in HFA (24%; *p* = 0.030), and group differences in left central beta ERD were statistically significant (27%; *p =* 0.046).

**Figure 5:**
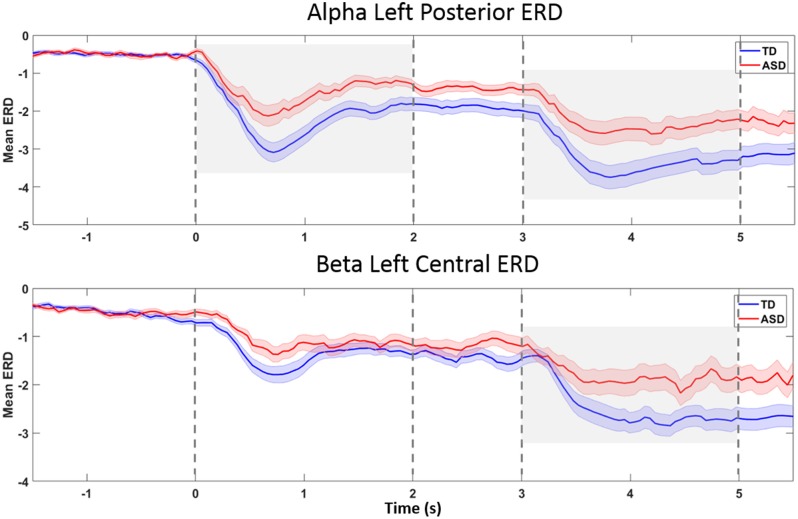
**ERD-over-time, in topographies and bands found to be different between groups.** The *x-*axis reflects time (in seconds), and the *y*-axis represents ERD magnitude (in unitless *z-*scores). The grand average for the TD group is plotted in blue, and the grand average for the HFA group is plotted in red. Shaded areas around the mean (heavy line) represent standard errors. The *Prepare* phase ERD measurement in statistical calculations is the average from 0 to 2 s on the *x-*axis, and the *Go* phase, from 3 to 5 s. Plot A represents left posterior alpha ERD. Group differences were seen during both the *Prepare* and *Go* phases (gray shaded areas). In left central beta ERD (Plot B), the *Go* phase ERD was significantly different between groups (gray shaded area). The plots suggest that a delay in onset of ERD or temporal dispersion is unlikely to explain group differences in average ERD.

The ERD showed similar time courses in both groups (Figure 5), suggesting that group differences in timing of praxis initiation or temporal dispersion (group differences in variability of timing across trials) are unlikely to explain measured group differences in mean ERD.

Because of significant group differences in the number of valid trials per subject (HFA = 86.12 ± 14.9; TD = 102.5 ± 11.2; *p* < 0.001), the above *t*-tests were conducted *without* the assumption of equal variances, as described in section “ERD Quantification”.

There were no statistical differences between groups in the baseline spectral power, in either ROI × either frequency band (see “Supplementary Data 3”).

### Behavioral Measures and Correlations with EEG

Children with HFA had significantly worse scores on the praxis performance battery (mean total percent correct = 74% for TD and 53% for HFA, *p* < 0.001), consistent with several previously published studies that included samples that did not overlap with that from the current study (Mostofsky et al., 2006; Dziuk et al., 2007; Dowell et al., 2009; MacNeil and Mostofsky, 2012). SRS data also, expectedly, differed between groups (HFA mean: 97; TD mean: 17.6; *p* < 0.001).

We correlated behavioral results (ADOS, SRS and pediatric FAB) with those ERSP measures that were different between groups. No statistically significant correlations were seen in the TD group. Within the HFA group, left central ROI beta ERD (during *Go*) correlated with praxis imitation performance (*r* = −0.4; *p* = 0.04). This left central beta ERD also correlated with ADOS total score (*r* = 0.48; *p* = 0.016). (See “Supplementary Data 4”). *Post hoc* analysis of ADOS sub-scores showed correlations between the same ERD measurement on one hand, and the Communications sub-score and Repetitive and Restricted Behaviors subscores of the ADOS on the other.

## Discussion

The primary finding of this research is decreased magnitude of task-related EEG power modulation in HFA during the performance of skilled movements on a praxis task—which is known to be impaired in ASD and has been shown to be associated with the core features of the disorder (Dziuk et al., 2007). These findings suggest that autism and its associated dyspraxia may be associated with decreased activity in the frontal-parietal praxis network.

Alpha activity was originally described as representing an idling state of the underlying cortical region (Pfurtscheller et al., 1996), however more recent accounts have demonstrated the role of alpha activity in inhibiting sensory input and/or maintenance of sensory-linked representations in primary sensory cortex (Kelly et al., 2006; Tuladhar et al., 2007; Ikkai et al., 2014). Suppression (ERD) of alpha activity therefore correlates with increased task-related activity of a cerebral region and differentiated the two groups. Decreased modulation of alpha in the parietal cortex suggests decreased task-related parietal activation in HFA.

Beta is a critical frequency in the motor system, having been documented not only in motor cortex, but in basal ganglia and muscle activity, as well as coherently among the parts of the motor system (Baker et al., 1999). Beta activity has been linked with the resting state of the motor system (Neuper et al., 2006) or with the maintenance of the current state of the motor system (Engel and Fries, 2010), while beta ERD has been linked with top-down motor control and with motor imagery (Neuper et al., 2006; Zhang et al., 2008; Engel and Fries, 2010). The current study showed that decreased central beta ERD during the praxis task correlates within the HFA group with praxis imitation ability and with severity of autism symptoms. Imitation has long been known to be impaired in ASD (DeMeyer et al., 1972; Williams et al., 2004), and it may be that deficiencies in the ability of the imagery and production system to activate in complex motor tasks underlies both impaired imitation and impaired complex gesture production.

In this experiment, we have demonstrated correlations between behavior and physiology. One challenge endemic in this type of research is determining which level of analysis drives the other. On one hand, it may be cognitive differences—in attention, motor imagery or event task performance—that drive physiological markers of task participation—ERSP. On the other hand, it may be altered neurobiological mechanisms—the ability to modulate cortical rhythms to respond to task demands—that produce behavioral group differences. We took pains to limit the influence of behavioral group differences on the EEG measures, by creating an easy task and by removing all behaviorally bad trials from EEG analysis, but it is impossible to rule out that there could be performance-related differences that were not quantified yet influenced the EEG responses. Unlike in research on lower-dimensional motor behaviors, it is difficult to find one or a few kinematic variables that adequately describe the movement, so as to control completely for behavioral differences.

The alternative view is that biological differences in the ability to modulate physiological rhythms in the brain are causally responsible for group differences in performance on the praxis task and other measures of motor and social/communicative differences. While the current data are not conclusive, converging data may bolster the latter view. First, a collection of results demonstrating an association between specific deficits in ASD and well-established behavioral effects of oscillatory modulation would provide evidence supportive of the proposition that the physiological effects are primary. The literature is in early stages, but a recent study of multisensory attention selection, an impaired ability in ASD, has demonstrated deficient alpha ERS (Murphy et al., 2014); given the putative role of alpha in sensory suppression (Kelly et al., 2006; Tuladhar et al., 2007), there is a compelling argument that deficits in alpha modulation *cause* alterations of multisensory attentional behavior. Further, future work with animal models, cellular physiology and computer modeling of pathological differences may establish the presence of physiological differences build up from knowledge of genetic mechanisms, without resorting to behavioral tasks. Finally, and perhaps most compellingly, interventional approaches with non-invasive brain stimulation may be able to perturb oscillatory activity sufficiently to draw direct inferences about the role of oscillations in behavioral differences in ASD (Helfrich et al., 2014). Each of these approaches has its limitations, but together, they would shed the most conclusive light on the purported role of altered oscillatory activity and neural synchrony in ASD (Uhlhaas and Singer, 2006; Uhlhaas et al., 2009), due to altered excitatory/inhibitory balance (Rubenstein and Merzenich, 2003) and the functional consequences of altered cytoarchitecture (Casanova, 2007).

Because this study is a novel examination of multidimensional physiological data (i.e., modulation of oscillations within the praxis network, in ASD), the analysis is necessarily somewhat exploratory. A further limitation is that we only examined children with *high functioning* autism. Lower-functioning children are worthy of study, and, indeed electrophysiological techniques hold the promise of providing mechanistic insights and biomarkers in children who are cannot be tested behaviorally because they are unable to make complex behavioral responses. In order to develop these biomarkers, however, we need to validate the link between EEG measurement and behavior in higher functioning children who are indeed able to make behavioral responses so as to validate the relevance of the physiological markers.

A few notes should be made about the extensive efforts made to reduce the likelihood of spurious group differences in the EEG data (Webb et al., 2015). First, the task contained both a motor execution phase (to monitor compliance with the task) and a motor planning phase (which would not be affected by differences in motion artifact). That group differences were seen during the *Prepare* phase limits the ability to ascribe group differences to motion artifact. Further, experimental effects were specific in topography and frequency, whereas many types of EEG artifact are symmetrical and broadband. Absence of group differences in the baseline spectra also suggests that artifact does not play a role in the ERD differences. Absence of baseline spectral differences also minimizes the likelihood that differences in overall cognitive state (e.g., attention) impacted ERD results. Further, as described above, we minimized the potential impact of performance-related group differences by creating a relatively easy task and removing trials (and subjects) with excessive errors. Since children with ASD may have differences in the timing of cortical events, we constructed both the task and analysis such that they are relatively insensitive to changes in timing of a relevant magnitude; additionally, we removed trials where responses were delayed or anticipatory. As described above, we constructed the stimuli to minimize the effects of potential group differences in reading and working memory ability. Finally, we controlled for potentially spurious alterations of SNR by equating the number of trials in the two groups.

In summary, children with HFA show a decreased ability to generate task-related changes in ongoing oscillatory activity during a praxis task. These group differences occurred in specific cerebral regions and frequency bands that are known to be relevant to the network involved. Electrophysiological differences correlate with autism symptom severity and with diagnosis-relevant differences in praxis function. These findings suggest that alterations in the mechanisms that generate cortical oscillations may contribute to known differences in praxis function and in the core aspects of autism.

## Author Contributions

JBE, BML, MH, NEC, and SHM contributed to the design of the experiments. ASP, DM, BML, and CN contributed to the acquisition of data. JBE, ASP, DMcA, and BML contributed to the analysis of data. JBE, ASP, DMcA, BML, MH, NEC, and SHM contributed to the interpretation of the data. All authors reviewed the manuscript for important intellectual content.

## Funding

This work was funded by the National Institute of Neurological Disorders and Stroke/National Institutes of Health (Intramural Program—MH; K23NS073626 to JBE; and R01NS048527 to SHM) and Autism Speaks (to SHM).

## Conflict of Interest Statement

The authors declare that the research was conducted in the absence of any commercial or financial relationships that could be construed as a potential conflict of interest.
