# Supplementary material for: Decreased Modulation of EEG Oscillations in High-Functioning Autism during a Motor Control Task
Source: Front Hum Neurosci. 2016 May 6;10:198. doi: 10.3389/fnhum.2016.00198 (PMC4858522; doi:10.3389/fnhum.2016.00198)
Supplement: Supplementary file 1 [file DataSheet_1.docx]

Supplementary Material

Decreased Modulation of EEG Oscillations in High-Functioning Autism During a Motor Control Task

**Joshua B. Ewen*, Balaji M. Lakshmanan, Ajay S. Pillai, Danielle McAuliffe, Carrie Nettles, Mark Hallett, Nathan E. Crone, Stewart H. Mostofsky**

*** Correspondence:** Corresponding Author: ewen@kennedykrieger.org

# Age-ERD Correlations

|  | **Left Parietal Alpha (*Prepare*)** | | **Left Parietal Alpha (*Go*)** | | **Left Central Beta (*Prepare*)** | | **Left Central Beta**  **(*Go*)** | |
| --- | --- | --- | --- | --- | --- | --- | --- | --- |
|  | rho | *p-*value | rho | *p-*value | rho | *p-*value | rho | *p-*value |
| **TD** | ***0.35*** | ***0.04*** | 0.31 | 0.082 | 0.037 | 0.84 | 0.080 | 0.66 |
| **HFA** | 0.23 | 0.27 | 0.26 | 0.21 | 0.071 | 0.77 | 0.22 | 0.30 |

In order to assess any potential effect of age on ERD, we examined correlations (Spearman’s rho). Only left parietal alpha during the *Prepare* phase, and only in the TD group, demonstrated group differences. Because the two groups were not different in age, it is improbable that group differences in ERD were driven by age-ERD associations.

# ANOVA TABLES

**2a. Alpha**

Within-Subjects Effects

|  | **Type III Sum of Squares** | ***df*** | **Mean Square** | ***F*** | **Sig.** | **Partial Eta Squared** |
| --- | --- | --- | --- | --- | --- | --- |
| **ROI** | 9.781 | 1 | 9.781 | 5.137 | 0.027 | 0.084 |
| ROI × Group | 11.466 | 1 | 11.466 | 6.023 | 0.017 | 0.097 |
| Error (ROI) | 106.617 | 56 | 1.904 |  |  |  |
| **Task** | 160.542 | 1 | 160.542 | 89.686 | 0 | 0.616 |
| Task × Group | 0.098 | 1 | 0.098 | 0.054 | 0.816 | 0.001 |
| Error (Task) | 100.242 | 56 | 1.79 |  |  |  |
| ROI × Task | 2.064 | 1 | 2.064 | 6.221 | 0.016 | 0.1 |
| ROI × Task × Group | 0.34 | 1 | 0.34 | 1.024 | 0.316 | 0.018 |
| Error (ROI × Task) | 18.577 | 56 | 0.332 |  |  |  |

Between-Subjects Effects

|  | **Type III Sum of**  **Squares** | ***df*** | **Mean Square** | ***F*** | **Sig.** | **Partial Eta**  **Squared** |
| --- | --- | --- | --- | --- | --- | --- |
| **Intercept** | 2157.435 | 1 | 2157.435 | 321.53 | 0 | 0.852 |
| **Group** | 16.921 | 1 | 16.921 | 2.522 | 0.118 | 0.043 |
| **Error** | 375.76 | 56 | 6.71 |  |  |  |

Tables show the results of the 2 x 2 x 2 repeated measures ANOVA with factors Task (*Prepare, Go*) and ROI (LP, LC) as the within-subjects factors and Group (ASD, TD) as the between-subjects factors for the Alpha band ERD data. There is a significant effect of ROI (*F*_1,56_=5.137, *p*=0.027, *η*_p_^2^ =0.084). There are also significant interactions between the factors ROI and Group (*F*_1,56_=11.466, *p*<0.017, *η*_p_^2^ =0.097). In addition, there is an effect of Task (*F*_1,56_=89.686, *p*<0.0001, *η*_p_^2^ =0.616), and an interaction between ROI and Task (*F*_1,56_=6.221, *p*=0.016, *η*_p_^2^ =0.1), which are significant.

**2b. Beta**

Within-Subjects Effects

|  | **Type III Sum of Squares** | ***df*** | **Mean Square** | ***F*** | **Sig.** | **Partial Eta Squared** |
| --- | --- | --- | --- | --- | --- | --- |
| **ROI** | 1.048 | 1 | 1.048 | 0.987 | 0.325 | 0.017 |
| ROI × Group | 2.506 | 1 | 2.506 | 2.36 | 0.13 | 0.04 |
| Error (ROI) | 59.478 | 56 | 1.062 |  |  |  |
| **Task** | 82.401 | 1 | 82.401 | 74.797 | 0 | 0.572 |
| Task × Group | 1.454 | 1 | 1.454 | 1.32 | 0.256 | 0.023 |
| Error (Task) | 61.693 | 56 | 1.102 |  |  |  |
| ROI × Task | 0.955 | 1 | 0.955 | 5.598 | 0.021 | 0.091 |
| ROI × Task × Group | 0.278 | 1 | 0.278 | 1.633 | 0.207 | 0.028 |
| Error (ROI × Task) | 9.55 | 56 | 0.171 |  |  |  |

Between-Subjects Effects

|  | **Type III Sum of**  **Squares** | ***df*** | **Mean Square** | ***F*** | **Sig.** | **Partial Eta**  **Squared** |
| --- | --- | --- | --- | --- | --- | --- |
| **Intercept** | 1138.685 | 1 | 1138.685 | 233.283 | .000 | .806 |
| **Group** | 11.329 | 1 | 11.329 | 2.321 | .133 | .040 |
| **Error** | 273.343 | 56 | 4.881 |  |  |  |

Tables show the results of the 2 × 2 × 2 repeated measures ANOVA with factors Task (*Prepare, Go*) and ROI (LP, LC) as the within-subjects factors and Group (ASD, TD) as the between-subjects factors for the Beta band ERD data. In the beta band, there is an effect of Task (*F*_1,56_=74.797, *p*<0.0001, *η*_p_^2^ =0.572) and an interaction between ROI and task (*F*_1,56_=5.598, *p*=0.021, *η*_p_^2^ =0.91), which are significant.

# Resting Power Group Comparisons

|  | **Left Parietal Alpha** | **Left Central Beta** |
| --- | --- | --- |
| ***p*-value** | 0.35 | 0.37 |
| ***t*-statistic** | -0.95 | -0.90 |
| **JZS Bayesian Factor** | 2.55 | 2.65 |
| **Information Bayesian Factor** | 1.91 | 0.984 |

We examined baseline (-2 to 0 sec relative to onset of the *Prepare* cue) power in the alpha and beta frequency bands as defined in the text, in those frequencies-topographies in which ERD group differences were seen. The data showed no significant group difference in EEG power.

# Scatter Plots of ERD-Clinical Correlations

**A:** Within the HFA group, magnitude of left central beta ERD correlates with autism severity, as measured by the ADOS-Total score. More negative ERD is more similar to that of controls; a higher ADOS score reflects more severe symptoms (*r* = 0.48; *p* = 0.016).

**B:** Within the HFA group, magnitude of left central beta ERD correlates with percent correct on the behavioral measure of praxis performance. More negative ERD is more similar to that of controls; a lower percent correct on the praxis task is more abnormal (*r* = -0.4; *p* = 0.04).
